# Diagnostic Accuracy Study of Near-Infrared Transillumination for Interproximal Dentinal Caries Detection in Primary Teeth

**DOI:** 10.3390/children13070890

**Published:** 2026-07-02

**Authors:** Andrea Armengol-Olivares, Laura Marqués-Martínez, Maria Carmona-Santamaría, Clara Maria Ferrer-Serrador, Irene Tormo-Gómez, Mónica Fernández-Mafé

**Affiliations:** School of Medicine and Health Sciences, Catholic University of Valencia, 46007 Valencia, Spain; laura.marques@ucv.es (L.M.-M.); maria.carmona@ucv.es (M.C.-S.); cm.ferrer@ucv.es (C.M.F.-S.); irene.tormo@ucv.es (I.T.-G.); monica.fernandez@ucv.es (M.F.-M.)

**Keywords:** dental caries, bitewing radiography, near-infrared light transillumination, NIRI, interproximal caries

## Abstract

**Background/Objectives:** Near-infrared light transillumination (NIRI) has emerged as a radiation-free adjunctive method for proximal caries detection in pediatric dentistry. However, evidence regarding its diagnostic performance in primary dentition remains limited. The aim of this prospective diagnostic accuracy study was to evaluate the diagnostic performance of NIRI for detecting interproximal dentinal caries lesions in primary molars, using bitewing radiography as a pragmatic clinical reference standard. **Methods**: Thirty-one pediatric patients aged 5–12 years were included, contributing 273 interproximal surfaces of primary molars. Clinical examination was performed using ICDAS criteria, while proximal lesions were assessed using bitewing radiography and NIRI integrated into the iTero Element 5D intraoral scanner. For diagnostic accuracy analysis, only dentinal lesions classified as D1–D3 according to the Mejàre classification were considered positive cases, whereas E0–E2 surfaces were classified as negative. Sensitivity, specificity, positive predictive value (PPV), negative predictive value (NPV), and overall diagnostic accuracy were calculated. **Results:** The overall prevalence of caries lesions was 37.4%; however, only 11 surfaces showed dentinal involvement (D1–D3) and were included as positive cases in the diagnostic accuracy analysis. NIRI demonstrated a sensitivity of 81.8% and a specificity of 74.8%. The PPV was 12.0%, whereas the NPV was 99.0%. Overall diagnostic accuracy was 75.1%. A total of 66 false-positive findings and 2 false-negative findings were identified. **Conclusions:** NIRI demonstrated high sensitivity and excellent negative predictive value for detecting interproximal dentinal caries lesions in primary molars, suggesting potential usefulness as an adjunctive screening tool. However, the low positive predictive value and high number of false-positive findings indicate that positive NIRI findings should be interpreted cautiously and should not be used as a standalone basis for treatment decisions. Further studies using more robust reference standards are required to clarify the clinical significance of NIRI-positive findings.

## 1. Introduction

Interproximal caries detection in primary dentition remains a diagnostic challenge in pediatric dentistry, particularly in areas with closed proximal contacts where direct visual examination is limited. Early identification of proximal lesions is important for implementing preventive and minimally invasive management strategies before cavitation progresses. Bitewing radiography (BWR) is currently considered the most widely used imaging modality for proximal caries detection and plays an important role in clinical decision-making, particularly for identifying lesions with dentinal involvement [1,2,3].

Although bitewing radiographs are routinely used in pediatric dentistry due to their accessibility and relatively low radiation dose, they present inherent limitations. Their two-dimensional nature may lead to anatomical superimposition, underestimation of lesion depth, and reduced sensitivity for early enamel demineralization [4,5,6]. Nevertheless, treatment decisions for proximal carious lesions are largely based on the radiographic assessment of lesion depth and, in particular, on the presence of dentinal involvement. One of the most widely used radiographic classification systems is that proposed by Mejàre et al. [7], which categorizes lesions according to the depth of radiolucency observed on bitewing radiographs (E0, E1, E2, D1, D2, and D3). This classification provides a detailed assessment of lesion progression, particularly within dentin, by dividing dentinal involvement into thirds rather than halves. Such subdivision allows a more precise characterization of early dentinal lesions, particularly in the D1 category, where cavitation is considered less likely according to Pitts and Rimmer [8]. Furthermore, repeated exposure to ionizing radiation should be minimized in pediatric patients according to the ALARA principle [9] and current recommendations of the American Academy of Pediatric Dentistry [10].

In this context, alternative non-ionizing diagnostic methods for proximal caries detection have gained increasing interest. Near-infrared light transillumination (NIRI), integrated into intraoral scanners such as the iTero Element 5D system, enables visualization of dental structures using near-infrared light without radiation exposure [11,12,13,14,15,16,17]. The technique is based on the different optical behavior of sound and demineralized dental tissues. Sound enamel demonstrates greater light transmission, whereas demineralized tissues scatter light due to structural porosity changes [18,19,20,21,22].

Previous studies have suggested that NIRI may be useful as an adjunctive diagnostic method for proximal caries detection, particularly for non-cavitated lesions [23,24,25]. However, evidence regarding its diagnostic performance in primary dentition remains limited, especially concerning specificity and false-positive findings. In addition, the clinical interpretation of NIRI-positive findings remains uncertain in the absence of histological validation.

Since dentinal involvement is considered clinically relevant for restorative treatment decision-making, evaluation of NIRI performance for detecting dentinal proximal lesions may provide clinically meaningful information regarding its potential role in pediatric dental practice.

Therefore, the aim of this prospective diagnostic accuracy study was to evaluate the diagnostic performance of near-infrared light transillumination for detecting interproximal dentinal caries lesions in primary molars, using bitewing radiography as a pragmatic clinical reference standard.

## 2. Materials and Methods

This prospective cross-sectional diagnostic accuracy study was designed and reported according to the STARD 2015 guidelines for diagnostic accuracy studies. The study protocol was approved by the Ethics Committee of the Catholic University of Valencia San Vicente Mártir (UCV/2023-2024/043) and conducted in accordance with the Declaration of Helsinki. Written informed consent was obtained from all parents or legal guardians prior to participation. Data collection was carried out between October 2023 and September 2024.

### 2.1. Inclusion/Exclusion Criteria

Pediatric patients aged 5 to 12 years who attended a pediatric dental clinic in Valencia were included in this study. Participant eligibility was assessed at the initial dental visit. The inclusion criteria were first-time attendance at the dental clinic, written informed consent provided by parents or legal guardians, the presence of closed proximal contact areas between primary molars and presence of at least one pair of primary molars with closed proximal contacts suitable for radiographic and NIRI evaluation. Exclusion criteria comprised the presence of physical, mental, or sensory impairments that could interfere with clinical examination, orthodontic appliances or space maintainers, hereditary enamel defects, and the absence of one or more primary molars due to agenesis or previous extraction.

### 2.2. Study Population

When a patient met the eligibility criteria, a member of the research team explained the purpose of the study to parents/legal guardians and invited them to allow the patient to participate anonymously and voluntarily, without any financial compensation. 

**Figure 1:**
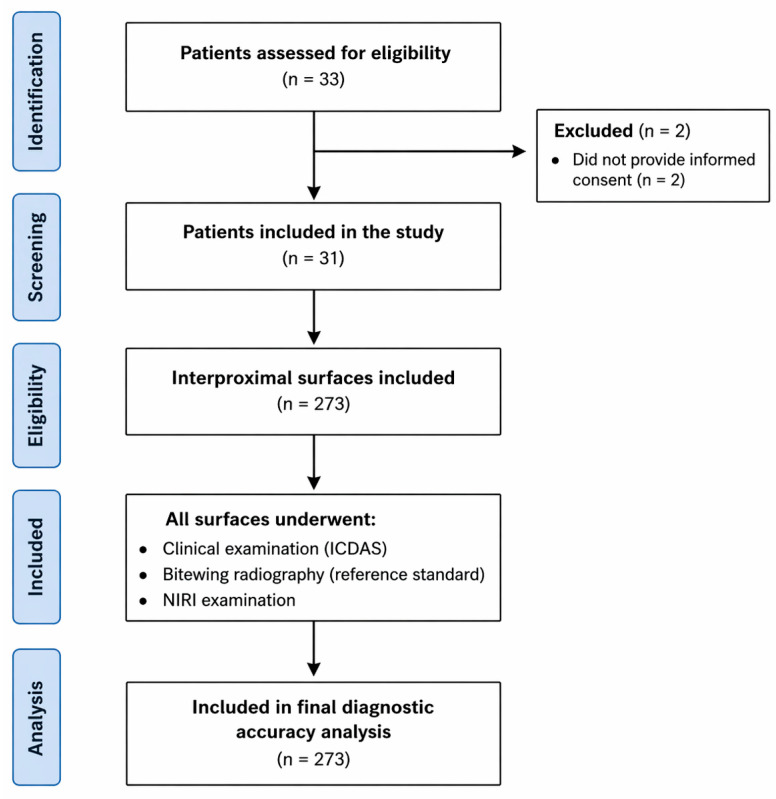
STARD flow diagram of patient inclusion and interproximal surface selection for diagnostic accuracy analysis.

A total of 33 pediatric patients were initially assessed for eligibility. Two patients were excluded due to the absence of signed informed consent from the parents or legal guardian. The final sample consisted of 31 children aged between 5 and 12 years (mean age: 8.0 ± 1.9 years), contributing 273 interproximal surfaces of primary molars for analysis (Figure 1).

The mean number of analyzed surfaces per child was 8.8 (range: 1–16). The evaluated surfaces included mesial and distal surfaces from primary molars (54, 55, 64, 65, 74, 75, 84, and 85).

### 2.3. Clinical Examination

Clinical examination of all participants was performed by two calibrated dentists under standardized conditions using a professional dental unit equipped with an operating light, compressed air, and a plane dental mirror.

For each participant, the following data were recorded: age (in months), sex (male or female), and number of erupted teeth. Clinical caries assessment was performed using the ICDAS classification system [26] to describe the presence and severity of caries lesions within the study population (Table 1). ICDAS findings were not used as the reference standard for diagnostic accuracy analysis. The reference standard for interproximal caries detection was based on bitewing radiography, given its routine clinical use for proximal lesion assessment in primary dentition. 

Clinical examination, bitewing radiography, and NIRI imaging were performed during the same clinical visit within a short time interval to minimize the risk of disease progression bias. No additional surfaces were excluded after enrollment except those not fulfilling the predefined inclusion criteria.

### 2.4. Radiographic Examination

Digital bitewings were taken with an HDX intraoral X-ray unit (Dental EZ, Lancaster, PA, USA); bitewing radiographs were obtained using photostimulable phosphor plates (PSP) and digitized using a VistaScan Mini Easy 2.0 system (Dürr Dental, Bietigheim-Bissingen, Germany) [27,28].The resulting digital images were displayed and evaluated under standardized viewing conditions. Radiographic interpretation was performed according to the Mejàre classification.

The rating of the BWR was done according to the Mejàre system Table 2 [7], as described below.

Experienced dentists reassessed all the images. If the examiners reached different conclusions, then they re-assessed the corresponding radiographs and discussed their points until a consensus was reached.

Bitewing radiography was used as the reference standard, as it is widely accepted in clinical practice for the detection of interproximal caries. However, it should be emphasized that bitewing radiography does not represent a true gold standard. Instead, it was considered a pragmatic clinical reference, given its routine use and its relevance for treatment decision-making. Its known limitations, particularly in detecting early enamel lesions, were considered when interpreting the results.

For the primary diagnostic accuracy analysis, only dentinal lesions classified as D1–D3 according to the Mejàre classification were considered positive cases. Sound surfaces and enamel-limited lesions (E0–E2) were classified as negative cases.

This threshold was selected because dentinal involvement is considered clinically relevant for restorative treatment decision-making and provides a more reproducible radiographic reference for diagnostic comparison.

### 2.5. iTero NIRI System

In order to perform the oral scan, the area was dried before starting image acquisition. An intraoral scanner iTero Element 5D (Align Technology, Inc., San Jose, CA, USA) equipped with near-infrared imaging (NIRI) technology was used [29]. Three-dimensional and infrared images were simultaneously acquired using the system’s proprietary software (iTero Scanner Software). Special attention was paid to ensure proper centering of each image over the region of interest, minimizing overlap of structures for each examined tooth.

The translucency of the scanned tooth structure to near-infrared light (NIRI, 850 nm) translates to the brightness level in the resulting captured image, wherein the higher the translucency of the object, the darker it appears. Intact enamel is translucent to NIRI and appears dark, whereas dentin and interferences in the enamel are reflective and scatter the NIRI, appearing brighter than intact enamel [11]. NIRI images from multiple angles are captured and stored automatically during the scan. Images were acquired and stored using the proprietary iTero software and were independently evaluated, blinded to clinical findings, by one calibrated examiner. The detection of proximal carious lesions was performed according to the classification proposed by Mejàre et al. [7]. For example, when a trapezoidal bright pattern detected on the NIRI image was confined to the amelodentinal junction without clear extension into the dentin, as observed on the mesial surface of tooth 5.5, the lesion was classified as D1. 

For the diagnostic analysis, NIRI findings were dichotomized as negative or positive. NIRI-negative corresponded to sound surfaces with no visible bright lesion pattern (score 0), whereas NIRI-positive corresponded to the presence of any caries-related bright lesion pattern classified as D1, D2, or D3 according to the Mejàre classification. Therefore, all lesions involving the amelodentinal junction or dentin were considered positive findings. All acquired NIRI images were of sufficient quality for interpretation; therefore, no images were excluded due to non-diagnostic quality.

### 2.6. Standardization, Calibration, and Blinding 

Prior to data collection, all examiners underwent calibration sessions for ICDAS assessment, bitewing radiographic interpretation, and NIRI image evaluation. Calibration consisted of theoretical training and practical exercises using extracted primary and permanent teeth presenting enamel and dentinal caries lesions.

Two experienced pediatric dentists independently evaluated the clinical examinations according to the ICDAS criteria and the bitewing radiographs according to the Mejàre classification. Inter-examiner agreement between the two pediatric dentists for bitewing radiographic scoring according to the Mejàre classification was assessed using Cohen’s kappa coefficient and demonstrated almost perfect agreement (κ = 0.90). In cases of disagreement regarding radiographic interpretation, the images were jointly re-evaluated until consensus was reached.

NIRI images obtained with the iTero Element 5D system were assessed by a third calibrated examiner who was blinded to the radiographic findings and ICDAS clinical evaluation. To assess intra-examiner reliability, a subset of NIRI images was re-evaluated by the same examiner after a six-week interval in randomized order. Intra-examiner agreement for NIRI interpretation demonstrated almost perfect agreement, with a Cohen’s kappa coefficient of 0.81.

The statistical analysis was performed by an independent investigator who did not participate in image acquisition or interpretation.

### 2.7. Sample Size Considerations

Data were collected using a consecutive non-probability sampling design. The unit of analysis was the interproximal surface, resulting in a total of 273 analyzed surfaces. 

Although no formal a priori sample size calculation was performed, confidence intervals were calculated for all diagnostic performance measures to improve interpretability of the results.

### 2.8. Statistical Analysis

Data were anonymized and analyzed using IBM SPSS Statistics version 23.0 (IBM Corp., Armonk, NY, USA). The primary analysis evaluated the diagnostic performance of NIRI using bitewing radiography as the pragmatic clinical reference standard. 

A 2 × 2 contingency table was constructed to calculate sensitivity, specificity, positive predictive value (PPV), negative predictive value (NPV), and overall diagnostic accuracy [30].

Inter- and intra-examiner agreement were assessed using Cohen’s kappa coefficient and interpreted according to the Landis and Koch classification [31].

Descriptive statistics were expressed as means and standard deviations for continuous variables and as absolute frequencies and percentages for categorical variables.

Because multiple interproximal surfaces originated from the same child, observations were not statistically independent. To account for within-patient clustering, 95% confidence intervals for sensitivity, specificity, positive predictive value (PPV), negative predictive value (NPV), and overall diagnostic accuracy were estimated using a patient-level cluster bootstrap procedure. In this approach, children rather than individual tooth surfaces were resampled, thereby preserving the correlation structure among observations within each patient.

A significance level of *p* < 0.05 was established for all analyses.

## 3. Results

### 3.1. Distribution of Surfaces and Caries Prevalence

The final sample consisted of 31 children aged between 5 and 12 years (mean age: 8.0 ± 1.9 years), contributing 273 interproximal surfaces of primary molars for analysis.

Of the analyzed surfaces, 154 (56.4%) belonged to female patients and 119 (43.6%) to male patients.

**Figure 2:**
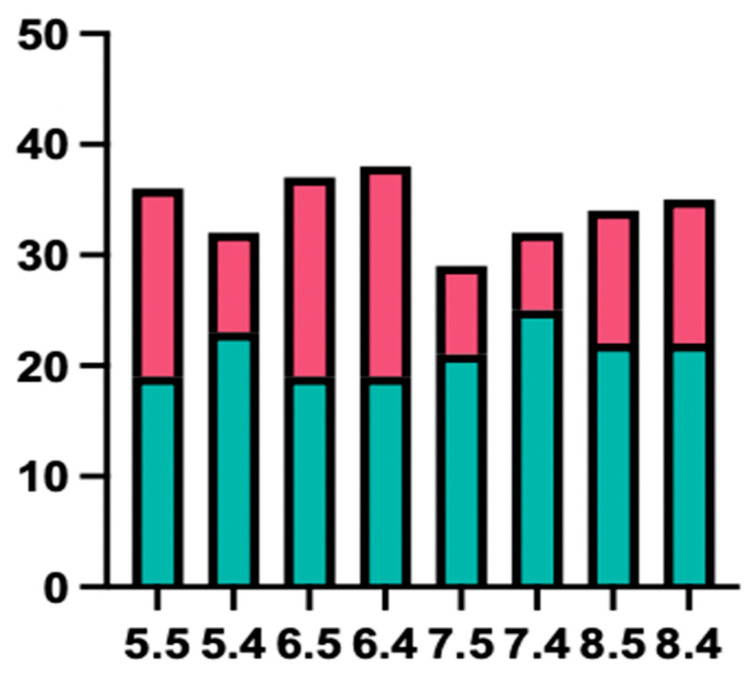
Distribution of sound and carious interproximal surfaces according to tooth type. Green bars represent sound surfaces, whereas red bars represent surfaces presenting carious lesions. Tooth numbering follows the Fédération Dentaire Internationale (FDI) system for primary dentition (54, 55, 64, 65, 74, 75, 84 and 85).

Of the 273 interproximal surfaces evaluated, 137 (50.2%) corresponded to mesial surfaces and 136 (49.8%) to distal surfaces. Overall, 102 surfaces (37.4%) presented caries lesions, whereas 171 surfaces (62.6%) were classified as sound. The distribution of carious and sound surfaces according to tooth type is shown in Figure 2.

### 3.2. Radiographic Distribution According to the Mejàre Classification

**Figure 3:**
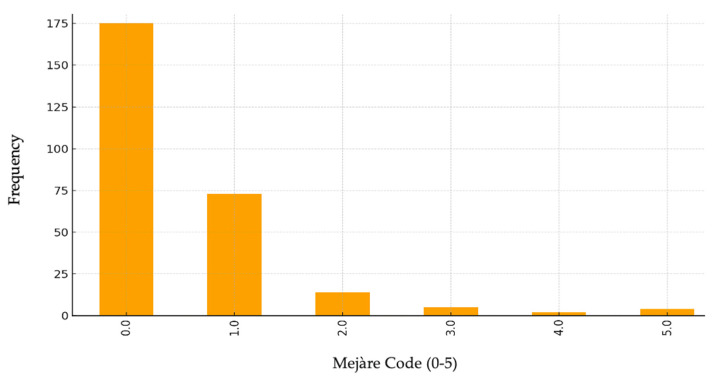
Distribution of interproximal surfaces according to the Mejàre radiographic classification (n = 273 surfaces). E0 = no radiolucency detected; E1 = radiolucency confined to the outer half of enamel; E2 = radiolucency extending into the inner half of enamel; D1 = radiolucency extending into the outer third of dentin; D2 = radiolucency extending into the middle third of dentin; D3 = radiolucency extending into the inner third of dentin.

According to bitewing radiographic assessment using the Mejàre classification, 175 surfaces (64.1%) showed no radiolucency (E0). Enamel-limited lesions (E1–E2) were identified in 87 surfaces (31.9%), whereas dentinal lesions (D1–D3) were detected in 11 surfaces (4.0%) (Figure 3).

Overall, the prevalence of any caries lesion (E1–D3) was 37.4% (102/273 interproximal surfaces). However, only dentinal lesions (D1–D3) were considered positive cases for the primary diagnostic accuracy analysis.

### 3.3. Clinical Evaluation Using ICDAS

**Figure 4:**
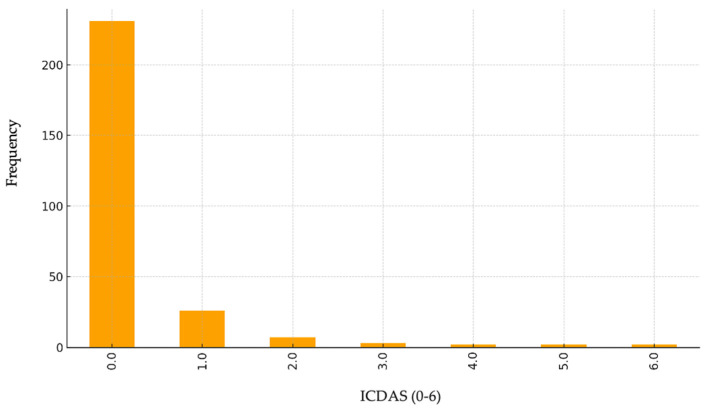
Distribution of ICDAS scores among the 273 evaluated interproximal surfaces. ICDAS scores range from 0 (sound tooth surface) to 6 (extensive cavitated lesion with visible dentin). Most evaluated surfaces corresponded to sound teeth or early non-cavitated lesions (ICDAS 0–2).

The distribution of surfaces according to the ICDAS clinical classification showed a clear predominance of initial lesions. A total of 55.3% of the surfaces (n = 151) were classified as ICDAS 0, followed by ICDAS 1 (23.4%; n = 64) and ICDAS 2 (10.3%; n = 28). Codes corresponding to cavitated lesions (ICDAS 3–6) accounted for a smaller proportion of the sample (11.0% overall), indicating that most lesions did not present visible clinical cavitation, probably related to the presence of closed interproximal contact points (Figure 4).

### 3.4. NIRI Findings and Diagnostic Accuracy

The NIRI system integrated into the iTero Element 5D scanner classified 75 surfaces (27.5%) as positive and 198 surfaces (72.5%) as negative for proximal caries lesions.

For the primary diagnostic accuracy analysis, dentinal lesions classified as D1–D3 according to the Mejàre classification were considered positive cases, whereas E0–E2 surfaces were classified as negative.

A total of 11 surfaces were classified as positive according to the reference standard and 262 as negative. The distribution of true-positive, false-positive, true-negative, and false-negative findings is presented in Table 3.

The NIRI system correctly identified 9 of the 11 dentinal lesions detected by bitewing radiography, resulting in 2 false-negative findings. Among the 262 radiographically negative surfaces, 196 were correctly classified as negative and 66 were classified as false positives.

Diagnostic performance measures are summarized in Table 4.

Cluster-adjusted confidence intervals were wider than those obtained under the assumption of independent observations, reflecting the correlation among multiple interproximal surfaces contributed by the same child.

The high negative predictive value suggests that NIRI may be useful for ruling out dentinal involvement, whereas the low positive predictive value indicates a substantial number of false-positive findings.

## 4. Discussion

The present study evaluated the diagnostic performance of near-infrared light transillumination (NIRI) for the detection of interproximal carious lesions with dentinal involvement in primary molars, using bitewing radiography as a pragmatic clinical reference standard. The findings demonstrated high sensitivity and excellent negative predictive value, indicating that NIRI may be useful for identifying proximal surfaces unlikely to present dentinal involvement. Conversely, the low positive predictive value observed suggests limited ability to confirm dentinal lesions when a positive NIRI finding is present.

Previous studies have reported favorable diagnostic performance of near-infrared transillumination for proximal caries assessment [32,33,34,35]. Lin et al. [33] and Schlenz et al. [34] described NIRI as a promising adjunctive diagnostic method with performance comparable to bitewing radiography under certain conditions. Consistent with these findings, only a small number of false-negative results were observed in the present study.

A notable finding was the substantial number of false-positive results, which contributed to the low positive predictive value. These NIRI-positive surfaces may represent early enamel demineralization not detectable radiographically, optical artifacts, or anatomical features such as marginal ridges. Similar observations have been reported by Stratigaki et al. [35], who described reduced specificity and an increased risk of false-positive findings when using near-infrared transillumination, particularly for enamel-level changes.

From a clinical perspective, NIRI may represent a useful radiation-free adjunct for screening and monitoring proximal surfaces in children. However, positive findings should not be interpreted as definitive evidence of dentinal involvement and should be considered alongside clinical examination and, when indicated, bitewing radiography.

Several limitations should be considered when interpreting these results. First, bitewing radiography was used as a pragmatic clinical reference standard rather than a histological gold standard, since histological validation was not feasible in vivo. Consequently, some surfaces classified as false positives may have represented early lesions not detectable on radiographs, potentially leading to an underestimation of NIRI specificity.

Second, the number of lesions with dentinal involvement was limited. Together with the absence of a formal a priori sample size calculation, this may have affected the precision of the diagnostic accuracy estimates, particularly for sensitivity and positive predictive value. Future diagnostic accuracy studies should incorporate formal sample size calculations based on expected disease prevalence and the desired precision of diagnostic estimates.

Third, the study population was recruited from a single university-based pediatric dental clinic and consisted exclusively of children attending their first visit. Therefore, the findings may not be fully generalizable to broader pediatric populations with different demographic or disease characteristics. In addition, prevalence-dependent measures such as positive and negative predictive values should be interpreted within the context of the study sample.

Finally, multiple interproximal surfaces were obtained from the same child, resulting in clustered observations. Although confidence intervals were recalculated using a patient-level cluster bootstrap approach, future studies should employ analytical methods specifically designed for hierarchical diagnostic data, such as generalized estimating equations or mixed-effects models. Furthermore, despite examiner calibration and blinding, some degree of operator-dependent variability cannot be completely excluded.

Future multicenter studies including larger and more diverse pediatric populations, a greater number of dentinal lesions, and more robust reference standards are required to further clarify the role of NIRI in the assessment of proximal dentinal caries in primary dentition. Moreover, studies assessing the cost-effectiveness, implementation feasibility, and clinical benefit of NIRI-equipped intraoral scanners in routine pediatric dental care are warranted, particularly considering the relatively high acquisition costs of these systems.

## 5. Conclusions

Within the limitations of this study, near-infrared light transillumination (NIRI) demonstrated high sensitivity and an excellent negative predictive value for the detection of interproximal carious lesions involving dentinal tissue in primary molars.

These findings suggest that NIRI may be a useful adjunctive screening tool for ruling out dentinal involvement in pediatric patients. However, the low positive predictive value and the considerable number of false-positive findings indicate that positive NIRI results should be interpreted with caution and should not be used as the sole basis for clinical decision-making.

From a clinical perspective, NIRI appears valuable as a screening modality: a negative result is highly suggestive of the absence of dentinal involvement. In contrast, positive findings are not diagnostic and require confirmation with bitewing radiography (BWR). The high rate of false positives highlights that reliance on NIRI alone could lead to potential overtreatment.

Further studies with larger pediatric samples and improved reference standards are required to better clarify the clinical relevance of NIRI-positive findings and to define its role in the detection of dentinal involvement.

## Figures and Tables

**Table 1:** ICDAS system chart.

CODING	CRITERION
0	Sound tooth surface
1	First visual change in enamel after prolonged air drying (white or brown opacity)
2	Distinct visual change in enamel visible on wet surface (white or brown lesion)
3	Localized enamel breakdown without visible dentin or underlying shadow
4	Underlying dark shadow from dentin with or without localized enamel breakdown
5	Distinct cavity with visible dentin, involving less than half of the tooth surface

**Table 2:** Mejàre system chart.

CODING	CRITERION
E0	No radiolucency detected
E1	Radiolucency confined to the outer half of enamel
E2	Radiolucency extending into the inner half of enamel, approaching the amelodentinal junction
D1	Radiolucency extending just beyond the amelodentinal junction into the outer third of dentin
D2	Radiolucency extending into the middle third of dentin, without approaching the pulp
D3	Radiolucency extending into the inner third of dentin, approaching or reaching the pulp

**Table 3:** Confusion matrix comparing NIRI findings with bitewing radiography according to the diagnostic threshold D1–D3.

NIRI Result	Bitewing Positive (D1–D3)	Bitewing Negative (E0–E2)	Total
Positive	9	66	75
Negative	2	196	198
Total	11	262	273

**Table 4:** Diagnostic performance of NIRI for detecting dentinal interproximal caries lesions using bitewing radiography as the reference standard. Confidence intervals were adjusted for clustering at the child level using a cluster bootstrap approach.

Diagnostic Parameter	n/N	Estimate (%)	95% CI ^1^
Sensitivity	9/11	81.8	50.0–100.0
Specificity	196/262	74.8	64.2–82.8
Positive predictive value	9/75	12.0	4.8–20.5
Negative predictive value	196/198	99.0	97.1–100.0
Diagnostic accuracy	205/273	75.1	65.2–82.9

^1^ 95% confidence intervals adjusted for within-patient clustering using a patient-level cluster bootstrap procedure.

## Data Availability

The datasets generated and/or analyzed during the current study are not publicly available due to ethical and privacy restrictions related to the protection of participant data but are available from the corresponding author on reasonable request.
